# Evaluation psychiatrique et psychologique du syndrome de Tako-Tsubo: à propos d’un cas

**DOI:** 10.11604/pamj.2017.27.70.12434

**Published:** 2017-05-30

**Authors:** Ghizlane Slimani, Hicham Lakbiri, Fatima Zahra Sekkat

**Affiliations:** 1 Clinique Universitaire de Psychiatrie Ar-Razi, Université Mohammed V de Rabat, Maroc

**Keywords:** Cardiomyopathie de Takotsubo, comorbidité, regulation émotionnel, Takotsubo cardiomyopathy, comorbidity, emotional regulation

## Abstract

La cardiopathie de stress ou Tako Tsubo est une pathologie cardiaque évoquant un syndrome coronarien aigu, avec signes électrocardiographiques, augmentation des enzymes cardiaques, mais où la coronarographie ne retrouve pas d'anomalie. Elle touche généralement des femmes ménopausées, dans les suites d'un stress intense. Le mode de début est variable, depuis la douleur angineuse le plus souvent jusqu'au tableau de choc cardiogénique. Le mécanisme physiopathologique exact de l'affection reste débattu. Plusieurs hypothèses sont invoquées, dont la plus crédible semble être un hyperadrénergisme brutal, lié au stress. Notamment il n'existe pas de consensus sur le traitement et la prévention. On peut alors s'interroger sur l'existence d'une pathologie psychiatrique sous-jacente ou une personnalité prédisposée et donc sur la place du psychiatre dans cette prise en charge.

## Abstract (en)

Stress cardiomyopathy or Tako Tsubo cardiomyopathy is a cardiac pathology evoking acute coronary syndrome characterized by electrocardiographic signs, cardiac enzyme elevation and no obstructive coronary lesions. It generally affects postmenopausal women and it usually occurs after periods of intense stress. Disease onset is widely variable, ranging from anginal pain (most common) to cardiogenic shock. Exact pathophysiological mechanism continues to be debated. Various hypotheses have been posited. Abrupt elevation of adrenaline levels appears to be the most credible. In particular, there is no consensus on treatment and prevention. Questions may then be asked about the existence of an underlying psychiatric pathology or a personality predisposition and, therefore, about the role of the psychiatrist in the management of this condition.

## Introduction

**Figure 1:**
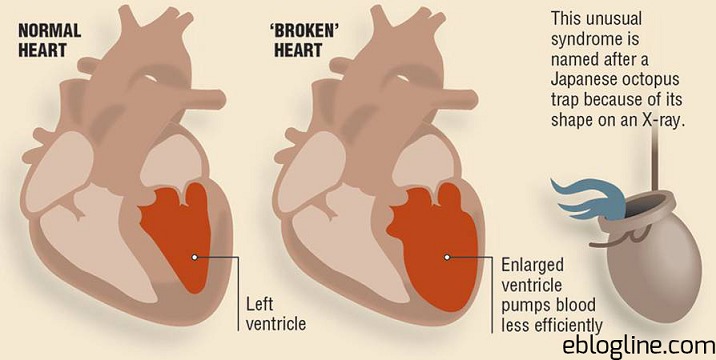
L’aspect du cœur dans la cardiomyopathie de stress (syndrome de Tako-Tsubo)

La cardiopathie de stress ou Tako Tsubo est une pathologie cardiaque évoquant un syndrome coronarien aigu marqué par une akinésie apicale ou médio-apicale du ventricule gauche avec signes électrocardiographiques, mais où la coronarographie ne retrouve pas d'anomalie (absence de sténose coronaire angiographiquement significative). Cette particularité de la contraction myocardique constatée à l'imagerie a donné le nom à ce syndrome dont la traduction littérale japonaise « tako»: poulpe «tsubo»: pot , correspond à un piège ancestral utilisé par les pêcheurs japonais dont la forme ressemble à celle du ventricule gauche pendant la systole (Figure 1) [1]. Cette cardiomyopathie de stress est beaucoup plus fréquente chez la femme que chez l'homme (de 80 à 100%), avec un âge moyen de début entre 61 et 76 ans, dans les suites d'un stress intense. Le mode de début est variable, depuis la douleur angineuse le plus souvent, jusqu'au tableau de choc cardiogénique. Le mécanisme physiopathologique exact de l'affection reste débattu. Plusieurs hypothèses sont invoquées, dont la plus crédible semble être un hyperadrénergisme brutal, lié au stress. Notamment il n'existe pas de consensus sur le traitement et la prévention. On peut alors s'interroger sur l'existence d'une pathologie psychiatrique sous-jacente ou une personnalité prédisposée, et donc sur la place du psychiatre dans cette prise en charge. Dans ce travail, à travers le cas de Mme Z, nous allons mettre en exergue les particularités de cette pathologie ainsi que les modalités et les difficultés de prise en charge.

## Patient et observation

Mme Z. âgée de 63 ans, ménopausée, suivie pour HTA depuis 13 ans sous inhibiteur de l'enzyme de conversion (IEC) bien équilibrée, opérée il y a 2 ans pour remplacement valvulaire mitral. Elle a été admise au service de cardiologie pour la prise en charge d'une douleur thoracique rétrosternale constrictive d'installation brutale, à irradiation scapulaire, suite à un stress intense. Un ECG a été réalisé aux urgences cardiologiques, objectivant une arythmie complète par fibrillation auriculaire (ACFA) et des ondes T négatives en V4-V5-V6 et en inferieur. Un bilan biologique a été demandé, revenu sans particularité avec un taux de troponine normal (0,187 ng /ml). Une échographie transthoracique a retrouvé une dysfonction ventriculaire gauche avec un fraction d'éjection (FE) à 38%, une akinésie diffuse et une fuite para-prothétique mitrale. La patiente a bénéficié par la suite d'une IRM cardiaque, qui a objectivé un VG non dilaté avec une dysfonction systolique FEVG à 37%, un OG ectasique, absence de rehaussement tardif, avec un aspect évocateur d'un syndrome de Tako-Tsubo. Mme Z. a été maintenue sous son traitement anticoagulant et antihypertenseur, puis adressée à l'hôpital psychiatrique Ar-razi pour complément de prise en charge. La patiente est issue d'un niveau socioéconomique modeste. Son enfance se serait déroulée sans particularité, sa scolarité était très moyenne arrêtée en première année du collège par désintérêt. Elle s'est mariée très jeune et a eu 6 enfants. Elle aurait mené une vie conjugale difficile avec son mari et sa belle-famille qu'elle qualifie d'autoritaires et très exigeants. Au fil des années elle serait devenue anxieuse avec soucis excessifs face au moindre problème de la vie quotidienne, avec des manifestations somatiques à type de transpiration, crampes musculaires, palpitations… A l'âge de 50 ans, une HTA a été découverte chez Mme Z.

Elle fut mise sous traitement antihypertenseur. 10 ans plus tard, elle fut opérée pour remplacement valvulaire, et c'est à ce moment-là que ses symptômes anxieux se sont exacerbés, avec un sentiment permanent d'insécurité. Elle serait devenue progressivement insomniaque, triste, anhédonique avec diminution de l'appétit. Elle serait restée sans aucune prise en charge psychiatrique, jusqu'au jour où elle aurait subi un stress intense (dispute violente avec un proche), et aurait présenté de façon brutale une véritable oppression thoracique avec une détresse respiratoire, ce qui a nécessité son hospitalisation au service de cardiologie. Après sa sortie, Mme Z. a bénéficié d'une consultation psychiatrique. L'entretien a objectivé un syndrome anxieux et un syndrome dépressif. Avant la mise sous traitement, elle a bénéficié d'un ensemble d'échelles d'évaluation psychiatrique (Tableau 1): le test de PSS 10 de stress perçu; Les échelles d'évaluation de dépression : MADRS, BDI et HAD de la dépression; Les échelles d'évaluation d'anxiété: Echelle de Hamilton et HAD d'anxiété; L'échelle d'obsession-compulsion de Yale-Brown (Y-BOCS); L'évaluation psychologique: test projectif de Rorschach et questionnaire de régulation émotionnelle (ERQ) (version arabe validée).

**Tableau 1:** les résultats des échelles d’évaluation psychiatrique

Échelles	Score minimal	Score maximal	Score obtenu	Interprétation
PSS 10	20	50	27	Il existe un certain nombre de situations qu’elle ne sait pas gérer, elle est parfois animée d’un sentiment d’impuissance qui entraine des perturbations émotionnelles.
MADRS	0	60	30	Présence de depression
BDI	0	39	15	Dépression modérée
HAD (D)	0	21	10	Dépression modérée
HAD (A)	0	21	7	Niveau d’anxiété dans la norme
Echelle d’Hamilton	0	56	17	Syndrome anxieux
Echelle Yale Brown	0	40	15	Très légère tendance obsessionnelle

**Évaluation psychologique:** La patiente a bénéficié d'un test projectif de Rorschach et questionnaire de régulation émotionnelle (ERQ) qui permet une évaluation du niveau de conscience, de compréhension et d'acceptation d'un individu envers ses états émotionnels. Il évalue deux processus: la suppression expressive et la réévaluation cognitive [2]; la suppression expressive inhibe les réponses émotionnelles mais souvent associée à des conséquences négatives: élévation des niveaux de stress et d'affect négatif et la détresse; la réévaluation cognitive, elle vise à amoindrir le caractère émotionnel d'une situation avant l'apparition du moindre élément émotionnel. Elle est associée à de meilleures conséquences psychologiques: mémorisation accrue des événements émotionnels ainsi moins d'anxiété ou de dépression; Le test de Rorschach a objectivé un défaut d'expression affective et fantasmatique, un excès d'investissement du réel dans ses détails avec un soucis d'objectivation obsédante. Aussi, il a mis en évidence des tendances dépressives qui la rendent inapte à diriger ses pensées avec une attention claire et un jugement exact. En ce qui concerne le test d'ERQ: Mme Z. a montré un niveau élevé du score de suppression (score = 24) et un faible niveau de réévaluation cognitive (score=18).

## Discussion

Le syndrome de Tako-tsubo est un dysfonctionnement ventriculaire gauche brutal dû à une augmentation soudaine des catécholamines circulantes consécutive à un stress majeur [3]. Le stress causal dans notre cas était représenté par la dispute violente avec un proche. Le lien de cause à effet entre la sidération apicale ventriculaire gauche et la mise en jeu massive du système adrénergiqentreue est solidement soutenu par le travail de Wittstein [4]. Ce dernier a trouvé une élévation des taux plasmatiques d'épinéphrine et de norépinephrine deux à trois fois aux niveaux observés chez sept patients atteints d'infarctus antérieur de classe III de Killip. Dans la littérature, on a trouvé que la prévalence des cardiomyopathies de stress pouvait aller jusqu'à 4,9% des syndromes coronariens chez les femmes [5]. Le profil principal est représenté par des femmes ménopausées, ayant des antécédents d'hypertension artérielle et porteuses de pacemaker, et qui ont fait face à des situations de stress. En ce qui concerne notre patiente, elle présente plusieurs facteurs expliquant la survenue de Tako-tsubo. Le premier est la ménopause, puisque la baisse de la sensibilité des barorécepteurs et de la réponse des adrénorécepteurs cardiovasculaires beta a été démontrée [6], ce qui pourrait expliquer l'élévation du rythme cardiaque et la vasoconstriction (spasme coronaire) durant un stress aigu. Le second facteur est la personnalité vulnérable de la patiente, révélée par le Test de Rorschach. Cette vulnérabilité est due à l'absence d'expression affective et fantasmatique, en plus de son incapacité d'adaptation face à une situation de stress physiologique ou psychologique, en raison de la rigidité de ses mécanismes de défenses, ce qui explique le score de PSS (=27). Quant à l'ERQ (questionnaire de régulation émotionnelle), Mme Z. a montré un niveau élevé de la suppression (= 24). Ces résultats montrent que la régulation émotionnelle de la personnalité de la patiente est basée sur la suppression, ce qui peut entrainer un terrain vulnérable après un évènement émotionnel stressant dans ce type de pathologie. À long terme, l'utilisation fréquente de la suppression expressive entraîne une diminution du contrôle de l'émotion et une symptomatologie dépressive et anxieuse [7], ce qui a été expliqué par les scores obtenus aux questionnaires de dépression (MADRS, BDI et HAD) et aux échelles de l'anxiété chez notre patiente. La présence de dépression dans ce type de pathologie a été rapportée dans plusieurs études: Vidi et al. (34 patients avec un tako-tsubo): 21% des patients présentent un trouble dépressif ou anxieux, ce facteur étant plus présent que d'autres facteurs de risque cardiovasculaires tels que le tabagisme ou le diabète [8]; Mudd et al.: ont retrouvé une prévalence du trouble dépressif ou anxieux de 40% chez 110 patients présentant un tako-tsubo [9]; Et plusieurs autres études ont objectivé des taux élevés de catécholamines, en particulier norépinephrine chez des patients dépressifs [10, 11].

Nguyen et al. ont retrouvé des résultats similaires (50% des patients avec un tako-tsubo présentent un trouble anxieux ou dépressif) en particulier le trouble panique. Dans le même sens, l'étude de Manfredini et al. (Série de 112 patients présentant un tako-tsubo) repère une distribution du tako-tsubo marquée par le jour de la semaine. Le nombre le plus élevé de cas de tako-tsubo concerne le lundi, le plus bas le samedi. Monfredini et al. font le lien entre anxiété et tako-tsubo en décrivant le lundi comme un jour particulièrement stressant (reprise du rythme de la semaine et de l'activité professionnelle) [12]. A noter que notre patiente a présenté un score de l'échelle d'obsession-compulsion de Yale-Brown (Y-BOCS) à 15 avec une très légère tendance obsessionnelle, ce qui est rapporté dans la littérature [13]. Il est possible d'émettre l'hypothèse que le niveau de suppression a contribué au maintien du symptôme dépressif après le premier traumatisme majeur (chirurgie de remplacement valvulaire), favorisant la vulnérabilité au Tako-tsubo via la diminution du tonus vagal et augmentation de la réponse hormonale adrénomédullaire aux événements stressants. Ce cas est concordant avec d'autres données disponibles [14] qui confirment l'importance physiopathologique de l'activation du système nerveux sympathique extrême dans ce type de maladie. Sur la base de nos résultats, il est raisonnable d'émettre l'hypothèse que l'origine de l'hyperréactivité sympathique peut être associée à certains traits émotionnels de la personnalité et à une suppression élevée.

## Conclusion

La prévalence de cardiomyopathie de stress est rare, et la physiopathologie de cette maladie reste à définir. Le cas que nous avons présenté met en exergue que l'état émotionnel pourrait prédisposer à ce type de pathologie. Cela implique que le risque d'un événement peut dépendre non seulement de la vulnérabilité cardiovasculaire de la personne suite à une exposition au stress, mais aussi de ses mécanismes d'adaptation.

## Conflits d’intérêts

Les auteurs ne déclarent aucun conflit d'intérêts.
